# Beyond Content Delivery: A Systematic Review of Video-Based SRL Interventions and Gaps in Explicit Motivational and Resource-Management Instruction

**DOI:** 10.3390/jintelligence14020033

**Published:** 2026-02-14

**Authors:** Anat Cohen, Orit Ezra, Efrat Michaeli, Guy Cohen, Hagit Gabbay, Alla Bronshtein

**Affiliations:** 1 School of Education, Tel Aviv University, Tel Aviv 6139001, Israel; oritezra@mail.tau.ac.il (O.E.); efratmich517@gmail.com (E.M.); guycohen@mail.tau.ac.il (G.C.); hg.astd@gmail.com (H.G.); 2 Tel Aviv University, Tel Aviv 6139001, Israel; allab@tauex.tau.ac.il

**Keywords:** self-regulated learning, video assisted learning, K-12, systematic review

## Abstract

Self-regulated learning (SRL) is a critical competency for learners in increasingly technology-enhanced educational environments, yet little is known about how SRL is fostered within video-based interventions in K-12 settings. While existing reviews and meta-analyses focus on the effectiveness of SRL interventions, this study aims to address current gaps by specifically examining the implementation processes, instructional tools, and the role of video. Addressing this, the present study conducted a systematic literature review of peer-reviewed K-12 intervention studies published since 2010, guided by PRISMA standards and other methodological frameworks in the field of SRL. 30 quantitative or mixed-methods studies focusing on K-12 SRL interventions were selected from Web of Science and ERIC, with the requirement that video served as an instructional component rather than a research tool. These studies were then systematically coded by eight researchers for SRL strategies, instructional methods, video roles, and pedagogical settings. Findings show that most video interventions targeted multiple SRL strategies across different phases of the SRL cycle, offering a comprehensive approach to fostering regulation. However, while cognitive and metacognitive strategies were frequently addressed, motivational and resource-management strategies were seldom included within explicit instruction, which focused primarily on cognitive and metacognitive training. Video played multiple pedagogical roles, including delivering disciplinary content, teaching SRL strategies, or combining both. A thematic analysis identified four pedagogical settings that characterized successful interventions: Teacher-guided, Active, Social, and Knowledge-based (TASK) learning. These settings appear to mitigate common challenges of video-based learning, such as cognitive load and learner passivity. The review contributes a novel synthesis of SRL-video integration and proposes TASK learning as a framework for designing SRL interventions.

## 1. Introduction

Self-regulated learning (SRL) involves a learner’s ability to control their learning process using metacognitive strategies (Zimmerman, 2000; Schunk & Zimmerman, 2023). It combines cognitive, metacognitive, and motivational strategies, enabling learners to effectively manage their learning, monitor progress, and maintain motivation (Zimmerman & Moylan, 2009). In our increasingly technological world, SRL is considered a fundamental skill for learners (Nilson & Zimmerman, 2023; Tsai et al., 2013).

Recent research has explored SRL from various angles. Theoretical studies have proposed different frameworks with similar foundations (Panadero, 2017), while empirical studies have investigated diverse strategies and sub-methods (Dignath et al., 2008). A common thread in these studies is the belief that SRL strategies can and should be taught (Callan et al., 2022; Pintrich, 1999).

A significant area of research focuses on intervention programs to promote SRL. These programs, which have gained momentum over the past two decades, aim to enhance learners’ SRL strategies either explicitly or implicitly. Explicit approaches involve direct instruction, modeling, or guided practice of SRL strategies. In contrast, implicit approaches embed opportunities for SRL development within learning activities or environments, such as cooperative learning, the flipped classroom, or project-based learning. These programs may be conducted by classroom teachers or researchers and are crucial in demonstrating that SRL can be effectively promoted through instruction and training. Research on these intervention programs examines their impact on various aspects of learning, including academic achievement, SRL strategy use, and motivation (Cousins et al., 2022; De Boer et al., 2018; Donker et al., 2014). These studies provide valuable insights into the effectiveness of different approaches to fostering SRL strategies, contributing to our understanding of how best to prepare learners for success in the modern educational landscape. Previous reviews and meta-analyses in the field offer a broad and nuanced understanding of intervention programs aimed at promoting SRL and their overall effectiveness. Nonetheless, a gap remains, as there has been no comprehensive, systematic investigation into how SRL is actually fostered within these interventions.

Furthermore, technology has introduced video-based learning as an educational tool, offering learners autonomy and flexibility (Shih & Huang, 2020; Van Laer & Elen, 2017). This method has gained popularity, especially since the COVID-19 pandemic (Delen et al., 2014) and the emergence of generative artificial intelligence (GenAI) (Zheng, 2025), becoming central to global education (Watters, 2023). Video-based learning environments are particularly relevant for promoting SRL as they combine high cognitive demands with learner-controlled pacing. As video unfolds dynamically over time, learners must actively monitor their understanding, decide when to pause or replay content, and regulate cognitive load, thereby engaging core SRL processes such as planning, monitoring, and regulation (Azevedo et al., 2015). Thus, video does not merely deliver content; it also implicitly functions as a context in which self-regulatory competence becomes both necessary and observable. Video can also be used to teach strategies explicitly drawing on its traits such as multimodal and short instruction (Sablić et al., 2021; Navarrete et al., 2025). Despite its importance and the need to foster SRL (He et al., 2016; Shih & Huang, 2020), research on integrating video to promote SRL strategies remains limited. Therefore, this study aims to present a systematic literature review of SRL intervention programs, focusing on how SRL strategies are trained and the role of video in these programs.

## 2. Current Literature Reviews and Meta-Analyses of SRL Intervention Programs

Existing systematic reviews and meta-analyses have sought to identify effective components, strategies, durations, and activities of SRL interventions. Examining these reviews revealed interesting insight regarding SRL interventions as follows: Synchronous intervention programs generally show success (Donker et al., 2014; Ergen & Kanadli, 2017), though opinions differ on the most effective strategies. Dignath and Büttner (2008) found that learners’ age influences outcomes: combined motivation and metacognition interventions were effective for primary students, whereas metacognition-based approaches were more effective for middle school students. Metacognitive knowledge instruction enhances intervention success (Ortube et al., 2024). Dignath et al. (2023) found that interventions that combined learning and behavior monitoring and encouraged metacognitive thinking were most effective.

A meta-analysis (Donker et al., 2014) shows that subject matter influences the effectiveness of SRL interventions. Some studies indicate that math interventions are most effective (Dignath & Büttner, 2008; Ergen & Kanadli, 2017), while others highlight the benefits of STEM subjects (Vilkova, 2022). Dignath and Büttner (2008) found teacher-led interventions more effective than researcher-led interventions, but De Boer et al. (2018) found the opposite, yielding conflicting findings in this area. Simón-Grábalos et al. (2025) reported that extracurricular interventions conducted by tutors or academic advisors faced challenges related to student motivation and participation. Finally, an umbrella review of systematic reviews and meta-analyses (Prasse et al., 2024) highlights the importance of adopting comprehensive approaches to the design and implementation of SRL supports. Specifically, these approaches integrate both explicit and implicit SRL supports across the entire SRL cycle.

### The Role of Video in SRL Intervention Programs

Technology can promote SRL (Azevedo et al., 2004), with video learning improving academic achievement (C. L. Lai & Hwang, 2016; Moos & Bonde, 2016), enhancing motivation, and engagement (Galatsopoulou et al., 2022). Video learning offers autonomy (He et al., 2016; Shih & Huang, 2020), allowing learners to control pace, revisit content, and choose learning time and location (Jansen et al., 2018). However, this independence necessitates strong self-regulation for success (Shih & Huang, 2020). Some existing literature reviews and meta-analyses have addressed the use of technology in SRL interventions, suggesting that video-integrated interventions are among the most effective technological tools (Vilkova, 2022), but their application varies widely (Delen et al., 2014; Van Alten et al., 2020a). Mejeh and Grieder (2025) suggested that a notable characteristic of effective interventions was the use of video analysis to enhance understanding of error analysis among vocational students.

Despite video’s significance in technology-aided learning (Watters, 2023), systematic reviews rarely focus on SRL interventions incorporating video. Addressing this gap, this study conducts a systematic literature review of K-12 SRL interventions, focusing on the instructional methods, learning tools, and pedagogical settings that characterized the interventions, as well as the role of video in these interventions.

## 3. The Study

Previous reviews and meta-analyses in the field provide a broad, in-depth understanding of intervention programs designed to promote SRL and their effectiveness. However, a gap remains in the existing body of knowledge, one that this systematic review seeks to address. Based on the existing reviews and meta-analyses presented above, to the best of our knowledge, there has not yet been a comprehensive, systematic examination of how SRL can be fostered. That is, a review of the implementation processes of interventions and the specific instructional tools employed, including training methods and scaffolds, is missing. In addition, existing literature reviews and meta-analyses on SRL interventions have not systematically examined the role of video in the studies they include.

Accordingly, this systematic review aims to address gaps identified in previous reviews and meta-analyses by conducting an in-depth examination of interventions that promote SRL through the integration of video in the K-12 system. The review seeks to understand what is being promoted, how it is promoted, the role of video, and what characterizes effective interventions. Therefore, this proposed review seeks to address the following questions:

Q1 Which SRL strategies do the interventions aim to promote?
Q2 Which instructional methods and learning tools do the interventions implement to promote SRL?
Q3 What are the roles of video within the SRL interventions?
Q4 What are the pedagogical settings that lead to a successful SRL intervention program?

## 4. Methodology

To address the research questions and ensure a rigorous systematic review process, we drew on two complementary methodological frameworks: the PRISMA standards (Page et al., 2021) and the review procedures outlined by Dignath and Büttner (2008), both of which were adapted to the context of the present study. Guided by these frameworks, the review proceeded in three main stages: (1) automatic retrieval of papers published from 2010 to 2023 across major databases (search date: March 2024); (2) researcher-based screening and construction of the relevant corpus; and (3) systematic coding of the variables in all eligible papers. The full research team, eight researchers, including doctoral and master’s students, participated in the first two stages, while four researchers conducted the coding stage. Notably, a formal methodological quality assessment was beyond the scope of the present review. The stages are detailed as follows:

### 4.1. Stage 1—Automatic Retrieval of Recent Papers (Published from 2010–2023) Based on Initial Criteria

**Figure 1:**
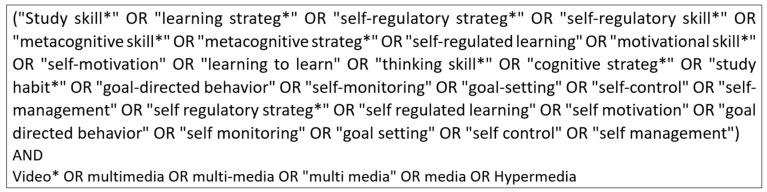
Search string used to retrieve the papers.

In this stage, we conducted searches using Web of Science and ERIC, two well-established databases with strong coverage of peer-reviewed research in education, learning sciences, and educational psychology. ERIC was selected for its focus on K-12 and educational research, whereas Web of Science provided broader interdisciplinary coverage, consistent with prior systematic reviews in the field. The search was based on keywords similar to those used by Dignath and Büttner (2008), such as study skill*, learning strateg*, and self-regulatory strateg*. In addition, video-related keywords were included, such as video*, *multimedia*, and multi-media*. Following is the string used to retrieve the papers (Figure 1):

The search was limited to English-language papers published in peer-reviewed journals or conference proceedings. More specifically, the search syntax was adapted to the indexing structures of each database. In ERIC, searches were conducted in the title, abstract, and descriptor fields, with filters applied for publication type (journal articles, speeches/meeting papers, and collection works-proceedings) and English language. In Web of Science, the search was conducted using the Topic field (including title, abstract, and keywords), with equivalent English language and document-type filters (proceeding paper and review article). The outputs from both databases produced a list of papers along with their metadata. We then identified and removed duplicate records, leaving us with 5890 papers. Finally, we retained only papers relevant to school contexts (K-12), following Dignath and Büttner (2008), by searching for the following keywords: *school*, *secondary*, *elementary*, *primary*, *junior-high*, *junior high*, *adolescent*, and *children* in the abstract and keyword fields. At the end of this stage, 1168 papers remained.

### 4.2. Stage 2—Construction of the Papers Corpus by the Research Team Based on Five Additional Criteria

To be included, a paper had to meet the following additional selection criteria: (1) The paper reports an intervention aimed at promoting SRL (at least one SRL strategy). (2) The paper’s study employs a quantitative (inferential or descriptive) or mixed-methods design. (3) The paper reports a school-based intervention (K-12). (4) The video plays some role in the intervention or the study reported in the paper. (5) The paper’s study was not using the video as a research tool (e.g., documenting the intervention on video). Each team member received approximately 150 papers to screen according to these criteria. A principal researcher was in charge of handling regular discussions with each team member including guiding each member on the selection criteria to employ while reading the abstract and full text and resolving discrepancies. The primary reason for exclusion at this stage was that many studies focused on higher education or other non–K-12 contexts. An additional primary exclusion reason was that many papers reported on correlative studies which did not involve an SRL intervention. These decisions were based on full-text examinations rather than abstract screening alone. Finally, 30 papers meeting the criteria were found. The list of papers included in the current literature review appears in Appendix A.

### 4.3. Stage 3—Variable Coding

To address the four research questions guiding the systematic review, four groups of variables were coded in Microsoft Excel for each paper in the corpus: (a) variables related to the SRL strategies that the interventions aimed to promote; (b) variables related to the instructional methods and learning tools that the interventions implemented to promote SRL; (c) variables related to the role of the video employed; (d) the pedagogical settings that lead to a successful intervention program. The principal researcher coordinated the coding process by holding regular discussions with team members, clarifying the four groups of variables, and resolving any discrepancies. The current literature review employs a mixed-method approach. Some of the research questions require a quantitative analysis of the data. Beyond that, there are sections where the answers to the research questions are open-ended.

**Figure 2:**
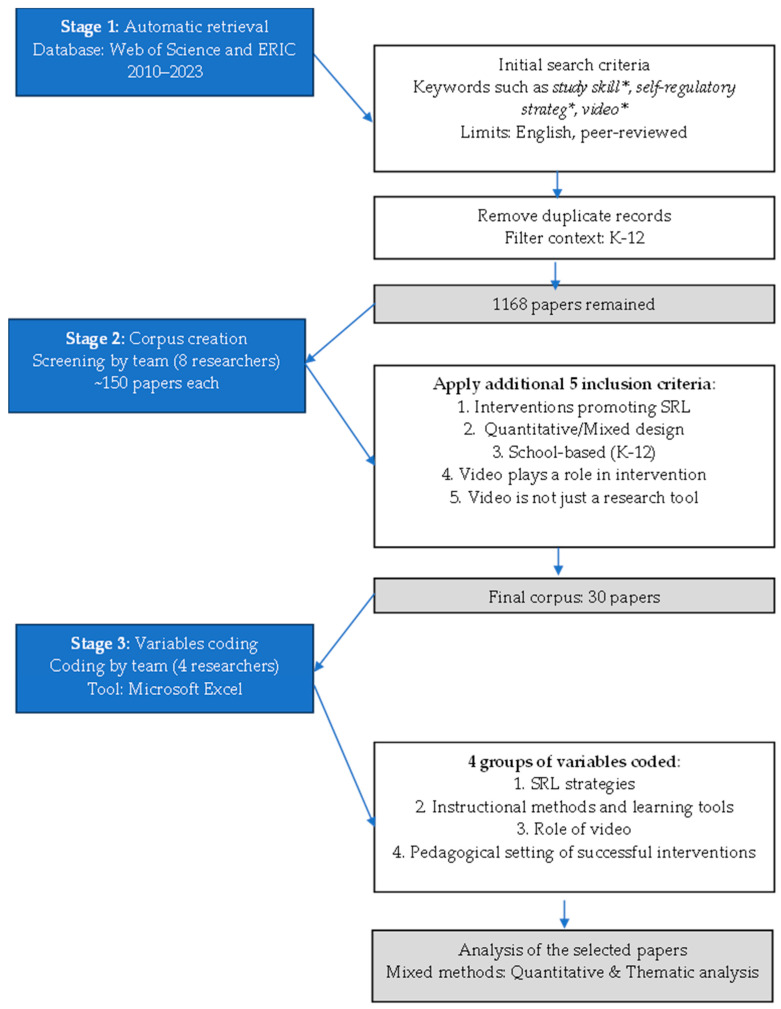
PRISMA flowchart visualizing the study selection process.

The PRISMA review protocol (checklist) (Supplementary Material) and the flow chart (Figure 2 below) were preregistered on the Open Science Framework (OSF) and are available at: https://doi.org/10.17605/OSF.IO/57MX6 (accessed on 9 December 2025).

## 5. Findings

A total of 30 papers met all eligibility criteria and were included in this review (see Appendix A). The selection process, which narrowed down the initial 1168 results through researcher-based screening, is summarized in the PRISMA flow diagram in Figure 2.

### 5.1. Q1 Which SRL Strategies Do the Interventions Aim to Promote?

The strategies that the interventions aimed to promote are listed in Appendix B. Most interventions aimed to promote cognitive strategies (e.g., problem-solving and note-taking) or metacognitive strategies (e.g., planning, goal setting, and reflection). In some cases, the interventions focused on a single strategy (e.g., papers 5 and 8), while others addressed several strategies (e.g., papers 3, 15, and 16), or even multiple strategies applied at different stages of the SRL process (e.g., papers 6 and 20). Breaking it down: 22 unique papers (73.33%) covered cognitive strategies, 19 (63.33%) meta-cognitive, 10 (33.33%) motivation, 7 (23.3%) resource management strategies.

Overall, the strategies targeted across the reviewed video interventions reflect core components identified in prominent SRL models, including cognitive processing, metacognitive regulation, motivational control, and management of learning resources. However, the distribution of strategies across these components was uneven, with a stronger emphasis on cognitive and metacognitive elements than on motivational and resource-management aspects. This finding suggests a potential gap between theoretical models of SRL and their practical implementation in video-based instructional design.

### 5.2. Q2 Which Instructional Methods and Learning Tools Do the Interventions Implement to Promote SRL?

The findings describe the instructional methods and learning tools that the interventions implemented to promote SRL, including their type (explicit/implicit) and their relation to the discipline. Table 1 presents a description of the instructional methods and learning tools used in the explicit and implicit interventions across the reviewed papers. The table outlines explicit instructional methods and learning tools, such as frontal strategy explanation and guidance using modelling, and implicit instructional methods such as inquiry learning, group work, gamification, and flipped learning environments. The table highlights the diversity of instructional methods and learning tools used to support SRL, in both explicit and implicit video SRL interventions.

21 papers used explicit instruction (70%), and 19 used implicit instruction (63.33%). 7 interventions (23%) taught strategies explicitly but also implicitly through other activities. 4 of these used explicit frontal instruction combined with implicit group work.

Table 2 organizes the papers by SRL strategy type (rows) and activity (columns), distinguishing between explicit and implicit instruction (the paper numbers are written in the cells). As can be seen in Table 2, there are 3 papers with two ways of teaching: two explicit SRL guided video interventions with prompts (paper 22), two implicit gaming and a learning environment (paper 21), two implicit flipped classroom and group work (paper 5). Looking at explicit instruction by strategy type, all explicit papers covered cognitive, meta-cognitive, or both; none covered motivation, and only two (6.7%) promoted resource management strategies. Implicit interventions covered all spectrum of strategies.

Regarding the relationship between the intervention and the discipline taught, some interventions are generic, while others are discipline-specific. In some cases, parts of the same intervention are generic, while others are discipline-specific. For example, in paper 2, the researchers combined discipline-specific problem-solving strategies in biology with generic strategies for task selection and evaluation.

In this context, three main points were revealed:

- Cognitive strategies were found in this review to appear both as specific and as generic. For example, papers 1, 2, 9, and 10 address cognitive strategies with a discipline-specific orientation, such as problem-solving in mathematics and biology, language-learning and reading comprehension strategies, and the promotion of abstract thinking skills in physics. In contrast, papers such as 6, 8, and 11 address generic cognitive strategies. Examples include promoting general critical thinking and problem-solving skills (e.g., classification, analysis, inference, consideration of multiple perspectives); fostering generic dimensions of critical thinking such as (1) identifying assumptions, (2) inductive reasoning, (3) deductive reasoning, (4) interpretation, and (5) argument evaluation; teaching generic reading strategies (annotations) ranging from basic comprehension of text and factual information to higher-level commenting on the text and inviting discussion; spaced practice, note preparation; and developing general problem-solving and persistence skills, for instance, “try solving it another way,” or “take a break and try again from a different starting point.”
- Metacognitive strategies are often associated with a generic orientation. For example, paper 6 deals with strategies such as planning (the preparatory phase), time management, structuring the learning environment, help-seeking, comprehension monitoring (the performance phase), and reflection.
- Many researchers refer to feedback in a generic orientation. However, feedback can also appear in a discipline-specific orientation. For example, in paper 7, the focus was on developing the ability to work with feedback in physical education, specifically concerning the correct technique for the Shot put.

### 5.3. Q3 What Are the Roles of Video Within the SRL Interventions?

The analysis revealed that video use can be divided into three main categories: videos that promote SRL, videos that contain disciplinary information, and videos that combine disciplinary information with SRL instruction through an additional layer of activity provided to the learner during viewing. These three main categories are further divided into different types of activities, as shown in Table 3. For each activity type, the table reports the proportion of papers that employed the approach and provides concise definitions that illustrate how the video functioned within the intervention. This structure highlights the diversity of video uses, ranging from explicit SRL instruction and video modelling to disciplinary content delivery, learner-generated video products that trigger the usage of SRL strategies, and multimodal environments that embed SRL scaffolding within subject-matter learning.

### 5.4. Q4 What Are the Pedagogical Settings That Lead to a Successful SRL Intervention Program?

The qualitative thematic analysis of the literature review, discussion, and conclusion sections of the selected papers revealed several pedagogical settings that can be described using the acronym TASK (Teacher-guided learning, Active learning, Social learning, Knowledge learning). These settings characterized successful video-integrated SRL interventions and illuminated the factors contributing to the effectiveness of the interventions. Successful video-integrated SRL interventions were assessed in the reviewed papers in various ways, for example, the use of SRL strategies, affective-emotional outcomes (e.g., motivation, satisfaction, confidence, self-efficacy), and cognitive outcomes such as performance and achievement.

Teacher-guided learning—teacher presence was identified as an important factor in six papers (20%). In general, the papers claim that teachers have an essential role in promoting students’ SRL, including in technology-enhanced environments (e.g., 15, 18). Some papers deal with students’ point of view, suggesting that students are seeking teachers’ help to promote their SRL (e.g., 14). Other papers deal with teachers’ beliefs. For example, paper 20 highlighted that teachers’ belief in the flipped classroom approach is crucial for student success, due to their ability to motivate learners and support their use of the model.

Active learning—inquiry-based learning and group work were frequently used to facilitate learner activeness and autonomy. Thirteen papers (43.3%) indicated that learners’ active engagement contributed to the success of the intervention. In paper 18, the group that did not participate in synchronous remote lessons but instead engaged through a video game demonstrated the greatest improvement in SRL across all experimental and control groups. This difference was attributed to the high level of learner activeness required by the game-based intervention. Similarly, paper 11 reported that higher levels of student participation were associated with better learning outcomes.

Several studies have highlighted learner autonomy as a central mechanism underlying active learning. Paper 17, which employed a virtual reality environment, emphasized that learners actively drove their own learning processes. In paper 19, increased autonomy was observed in an intervention that promoted reflective thinking through a personal learning management system. Likewise, paper 7 reported gains in learner autonomy following the SRL intervention. These findings point to a reciprocal relationship in which autonomy supports the development of SRL, while SRL, in turn, enables more effective autonomous learning. Consistent with this pattern, paper 20 explained how the flipped classroom model fosters SRL by structuring learning in ways that require learners to take responsibility for their learning and regulate their learning pace.

Social learning—emerged as a component of success in ten papers (33.3%). Peer-based activities, such as writing annotations and viewing each other’s notes, as well as sharing learning objectives and strategies (e.g., 12), were found to improve both achievement and SRL. The social component also served as a source of motivation, thereby further enhancing learning (e.g., 15, 18).

Knowledge-based learning—four papers (13.3%) identified prior knowledge as a key factor in intervention success. For example, prior knowledge was examined as an independent variable influencing student engagement and, by extension, achievement (8). Learners in a self-paced programming environment were provided with access to a “toolbox” button that allowed them to review prerequisite knowledge, based on the assumption that learners can appropriately apply SRL strategies only when they possess the necessary prior knowledge (14).

## 6. Discussion

In light of the lack of a comprehensive and systematic examination of how SRL can be fostered and the role of video in existing reviews and meta-analyses, this systematic review addresses this gap. It conducts an in-depth examination of interventions that promote SRL through the integration of video in the K-12 system. To do so, we examine what is being promoted, how it is promoted, the role of the video, and the successful pedagogical settings. The findings related to the research questions are discussed below.

### 6.1. SRL Strategies the Interventions Aim to Promote (Q1)

Many of the video interventions in this review sought to promote a combination of strategies across the SRL cycle, offering a comprehensive view of the learning process. This aligns with previous reviews recommending that by teaching the three SRL stages, complementary components will improve the effectiveness of the intervention programs (Dignath & Büttner, 2008; C. L. Lai & Hwang, 2023) and that diverse cognitive, meta-cognitive, and motivational strategy training is the most effective intervention (Dignath et al., 2008). This contrasts with Van Alten et al.’s (2020a) review, which found that multiple-strategy instruction overwhelmed learners.

More specifically, although previous reviews suggested a focus on problem-solving and elaboration (Dignath et al., 2008), the video interventions addressing cognitive strategies did not adequately incorporate these strategies. This may be explained by the fact that problem-solving is a relatively long and complex process (Cohen & Cohen, 2024), making short video-based instruction less suitable for supporting it, and by the possibility that elaboration is an internal cognitive process that is difficult to facilitate through video. In the area of metacognitive strategies, our findings show that video-based interventions can support planning strategies, as recommended by previous reviews (Dignath et al., 2008).

Most importantly, the findings show that the distribution of strategies was uneven, with a stronger emphasis on cognitive and metacognitive strategies than on motivational and resource-management strategies. This “Cognitive Bias” in SRL interventions is documented in several key meta-analyses and theoretical reviews (e.g., Dignath & Büttner, 2008). The long-term nature of motivational changes (Panadero, 2017) may explain the limited emphasis on motivational strategies. Resource management strategies have been less addressed in the literature, perhaps because traditional classroom settings often involve the teacher externally regulating resources. This approach should be reconsidered in video-based learning environments due to students’ ability to autonomously and actively engage in the learning process (Broadbent & Poon, 2015).

### 6.2. Instructional Methods and Learning Tools Implemented in the Interventions to Promote SRL (Q2)

Previous reviews and meta-analyses (e.g., De Boer et al., 2018; Donker et al., 2014; Simón-Grábalos et al., 2025) have not examined explicit versus implicit instruction in SRL. This review revealed a relatively balanced coverage of explicit and implicit instruction in SRL interventions, with some programs combining both approaches. Thus, some SRL video interventions included in this review adhered to Carter et al.’s (2020) recommendation to combine explicit and implicit methods to reduce cognitive load.

Examining how different instruction types addressed various strategy types in the video SRL interventions also revealed interesting findings. Notably, most video interventions do not explicitly promote motivational and resource management strategies. However, practical implicit approaches such as gamification, group work, and the flipped classroom are used to enhance motivational and resource management strategies. Therefore, a discrepancy exists between the literature and interventions, as the former emphasizes the importance of these strategies (Boekaerts, 2011) and highlights self-efficacy, task value, and intrinsic motivation (Carter et al., 2020). This discrepancy suggests a need to bridge the theory–practice gap. This raises questions about the feasibility of explicit motivational and resource management interventions and whether implicit methods are more suitable for instilling them. Thus, this study highlights the need for further research into explicit motivational and resource management SRL interventions.

Regarding the relation between the intervention and the discipline taught, this review found that video SRL interventions employed both generic and discipline-specific strategies, in some cases combining both types within the same intervention. Interestingly, cognitive strategies, which are often associated with a discipline-specific orientation (Dignath & Büttner, 2008; Dignath et al., 2008; Sijmkens et al., 2023), were found in this review to encompass both discipline-specific (e.g., language-learning reading comprehension) and generic (e.g., classification) strategies. Metacognitive strategies were often associated with generic orientation. Given that interventions combining various SRL strategies within subject-specific instruction have been found to yield significantly higher achievement (Cousins et al., 2022), video-based SRL interventions can be adapted for subject-specific instruction. This can be achieved by incorporating both discipline-specific strategies and generic strategies tailored to the tasks at hand.

### 6.3. Roles of Video Within the SRL Interventions (Q3)

There are multiple pedagogical avenues for integrating video into teaching and learning. The most prominent and widely documented use is the employment of video as a medium for delivering disciplinary content (Girón-García & Fortanet-Gómez, 2023; He et al., 2016), a pattern that was also reflected in the present review. The use of video to foster SRL has likewise been noted in previous research (Jansen et al., 2020), and this function also emerged in the interventions examined here. A further, though less common, approach involves having learners produce videos themselves, an activity that requires them to actively apply the SRL strategies taught. This mode of use was relatively rare in the current review, consistent with a recent systematic review of SRL-supportive technologies (Yang et al., 2023).

Although video conferencing is a central component of contemporary learning environments (Maulana, 2023), its use specifically for teaching SRL strategies was less prevalent in the studies included in this review. Another notable category identified involves videos that integrate disciplinary content with SRL instruction. The findings from this review indicate that such integration can be achieved through several pedagogical mechanisms, with prompts (K. T. Wong et al., 2019) representing the most common approach. At the same time, the increasing adoption of augmented and virtual reality technologies has given rise to new video-based models that combine gamification elements, domain-specific content, and explicit SRL instruction.

### 6.4. Pedagogical Settings That Lead to Successful SRL Intervention Programs (Q4)

The thematic analysis of the narratives of the included studies suggests that SRL interventions described as successful (based on reported strategy use as well as cognitive and affective outcomes) are often associated with teacher-guided, active, social, and knowledge-based (TASK) learning. *Teacher presence* is particularly important, as video-based learning is typically an individual, solitary activity, and young learners require human guidance to manage the cognitive demands associated with SRL (Carter et al., 2020). An *active learning* approach can further mitigate the limitations of passive video viewing; grounded in constructivist and social learning theories (Paas, 2014; Amineh & Asl, 2015), such approaches enhance intrinsic motivation by providing learners with greater autonomy (Chiu, 2022). *Social learning* can reduce cognitive load that may arise from the multimodal nature of videos, as peers attending to different information channels can complement one another’s processing. *Knowledge-based learning* can address the inherent limitations of short instructional videos, which often omit important background information (Sablić et al., 2021; Navarrete et al., 2025), by incorporating mechanisms that direct learners to additional learning resources.

### 6.5. Conclusions

This systematic review offers a comprehensive, integrative analysis of video-based SRL interventions in K-12 education, addressing a gap in existing reviews and meta-analyses that have rarely examined *how* SRL is fostered and the role of video within intervention programs. The findings suggest that video-based SRL interventions associated with positive outcomes frequently include pedagogical settings that align with teacher-guided, active, social, and knowledge-based (TASK) learning. These settings appear to mitigate limitations inherent to video learning, such as cognitive load, passivity, and reduced interaction, thereby illuminating why some interventions succeed while others remain less impactful.

The review also contributes to SRL scholarship by demonstrating that video-based interventions tend to promote multiple SRL strategies across the SRL cycle, often combining cognitive and metacognitive strategies. In contrast, motivational and resource-management strategies are less frequently addressed explicitly. Furthermore, the findings enhance our understanding of the diverse roles of video, ranging from disciplinary instruction to SRL scaffolding and mixed approaches. Together, these insights broaden the conceptual and practical landscape of SRL intervention design, underscoring the potential of video to support SRL.

Despite the contributions of this systematic review, several limitations should be acknowledged. First, the review focuses on K-12 student populations. Expanding future research to include other populations could yield a more comprehensive understanding of how video-based SRL interventions operate across different educational contexts. Further, although multiple K-12-related keywords were used to screen abstracts and keywords, and all retained articles were subsequently reviewed manually to verify their educational context, it is possible that some relevant K-12 studies were not identified due to variability in how educational levels are reported across publications. Moreover, although the paper screening and variable coding were guided by a clearly defined scheme and involved iterative discussions among the research team, no quantitative consistency measure was calculated. As a result, the reported counts and classifications should be interpreted with appropriate caution. Second, as video technologies continue to evolve (e.g., GenAI-driven adaptive video), future reviews should revisit the field to capture emerging forms of video-based SRL. Finally, the current review calls for further examination of the processes underlying successful SRL video integration, including systematic quantitative reviews of learner cognition during video viewing and the interaction between SRL strategies and video design features.

This review advances the field by offering the first systematic synthesis that simultaneously examines what SRL strategies are promoted, how they are taught, how video functions within interventions, and which pedagogical settings characterize successful programs. By introducing the TASK framework (Teacher-guided, Active, Social, and Knowledge-based learning) and highlighting emerging patterns across the literature, the review provides a conceptual lens to guide researchers and practitioners in designing SRL interventions. Furthermore, the review highlights areas where theory-practice gaps persist, particularly in motivational and resource-management instruction, thereby pointing to concrete directions for innovation in SRL pedagogy.

## Figures and Tables

**Table 1:** Types of explicit and implicit SRL instruction across the reviewed papers.

Explicit instruction	Frontal	Explicit strategy explanation involves demonstrating the steps, the facilitator’s implementation, questions, and class discussion. This is followed by group/individual practice of the explicitly taught strategy. For example, synchronous video instruction in paper 13 is classified as frontal due to its similarities with in-class learning, such as the inability to rewind the video and the option to get teacher feedback.
	Guidance	Similar to frontal instruction, video guides explicitly explain a strategy, its use, and rationale. Video Modeling (VM) documents an “expert” using a strategy, demonstrates its implementation through a think-aloud, and serves as an example. Another video-guiding method is an augmented reality system used for teaching abstract scientific thinking (paper 9).
	Prompts	Prompts are textual aids that appear to learners during regular work/tasks within a learning environment or in informational videos. Some prompts are preset with fixed timing, while others adapt based on log-file analysis of learner data. In paper 22, for example, prompts were provided in the form of memory-supporting strategy sheets.
	SRL online platform	Some learning environments promote SRL without explicit strategy instruction, but clarify their purpose of helping students implement SRL strategies alongside disciplinary learning. Activities include goal setting, self-assessment, note-taking, and required cognitive strategies like information gathering/organization. In paper 13, for example, learners prepare mind maps from self-collected materials, receiving task instructions that implicitly guide strategy implementation. These environments may have collaborative peer assessment/commenting components. Some use digital strategy implementation guides instead of pop-up prompts.
	Gamification	One intervention developed a VR educational game for explicit vocabulary learning.
Implicit instruction	Online learning environment	Some existing learning environments and learning management systems were not designed to explicitly promote SRL, but do implicitly facilitate the development of SRL strategies among learners.
	Gamification	Gamification implicitly facilitates strategy acquisition by embedding learning within educational games. In studies that incorporated gamification elements for learning, the aim was to increase learners’ motivation and engagement.
	Inquiry	Inquiry learning, often in groups, promotes learner freedom and autonomy. It involves searching for questions or problems, exploring topics, and generating solutions, as outlined in paper 15. This approach implicitly fosters SRL strategies for effective learning.
	Group work	Group work was widely used. Some used it solely, assuming SRL strategies could develop through social learning. Other interventions combined individual and group learning to deepen understanding or facilitate peer assessment. However, in 17 interventions, group work was not the main method used to teach the strategy; rather, it was used for SRL practice/application.
	Flipped classroom	The flipped classroom method, explored in three papers, involves students encountering material before class, often through pre-class videos. This allows for a deeper understanding in class through group work or by asking the teacher questions. With learners responsible for acquiring knowledge independently, SRL strategies become crucial.

**Table 2:** SRL strategies by implicit and explicit instruction type.

	Explicit Instruction					Implicit Instruction				
Activity\ Strategy Type	Frontal	Guidance	Prompts	SRL Online Platform	Gamification	Online Learning Environment	Gamification	Inquiry	Group Work	Flipped Classroom
Cognitive	3, 5, 13, 16	1, 2, 9, **22**, 24	6	8, 14	17	**21**, 24, 25	** 21 **	15	4, 12, 16, 29	20
Meta-cognitive	13, 3, 11	1, 2, **22**	6, **22,** 26	7, 12, 27, 30		19, **21**	18, **21**	15	4, 13, 29	6, 20
Motivational						23, 19	18	15	3, **5**, 8, 12, 29	20
Resource management			6	12		** 21 **	** 21 **	15	4	**5**, 6, 20

**Table 3:** Video roles in SRL interventions.

**Role**	**Activity Type**	**Papers (%)**	**Definition and Use**
Provide content for promoting SRL	Instruction	3 (10%)	The videos contain an explicit explanation of the strategy, how to use it, the reason for using it, and examples of its implementation.
	Video Modeling (VM)	3 (10%)	Instruction through a demonstration by an expert showing how to implement a specific strategy—not talking about it, but applying it. A recorded board is used, sometimes with think-aloud.
	Synchronized meeting	3 (10%)	In a synchronous meeting, video conferencing technologies were used, and learners met with the teacher in a ‘live broadcast’.
Provide disciplinary content	Instruction	10 (33.3%)	Videos for teaching the subject area were used to increase learners’ interest, reinforce prior knowledge, and introduce new material. Some videos were recorded by the teachers themselves, while others were found online and utilized by the teachers.
	Active search online	3 (10%)	The interventions combined the learners’ creation of a digital product, requiring them to search the internet for videos related to the activity topic.
	Video making	1 (3.3%)	Learners were asked to learn and research the subject area and integrate it with the use of SRL strategies in order to produce a video addressing social issues.
	Synchronized meeting	4 (13.3%)	The synchronous meeting was held to explain the domains.
Combining disciplinary and SRL content	Applying SRL	7 (23.3%)	Common approaches included SRL-promoting prompts during instructional videos, video-based games for subject/SRL learning, an AR environment for cognitive strategy acquisition alongside material exposure, and a 3D ‘digital expert’ guiding SRL based on learner performance.

## Data Availability

The raw data supporting the conclusions of this article will be made available by the authors on request.

## Supplementary Materials

The following supporting information can be downloaded at: https://www.mdpi.com/article/10.3390/jintelligence14020033/s1, Table S1: PRISMA 2020 checklist.

## Appendix A. Video-Based SRL Intervention Papers Included in the Current Literature Review

The full references of the 30 papers are marked with asterisks in the bibliography.

**#**	**Authors, Publication Year**	**Paper Name**
1	Raaijmakers et al. (2018)	Training self-regulated learning skills with video modeling examples: Do task-selection skills transfer?
2	Kostons et al. (2012)	Training self-assessment and task-selection skills: A cognitive approach to improving self-regulated learning
3	Lau (2020)	The effectiveness of self-regulated learning instruction on students’ classical Chinese reading comprehension and motivation
4	Jena et al. (2018)	Exploring the Effects of Web 2.0 Technology on Individual and Collaborative Learning Performance in Relation to Self-Regulation of Learners
5	T. L. Lai et al. (2020)	The Effectiveness of Team-Based Flipped Learning on a Vocational High School Economics Classroom
6	Van Alten et al. (2020b)	Self-regulated learning support in flipped learning videos enhances learning outcomes
7	Kok et al. (2020)	The effects of self-controlled video feedback on motor learning and self-efficacy in a Physical Education setting: an exploratory study on the shot-put
8	Chen et al. (2020)	A web-based collaborative reading annotation system with gamification mechanisms to improve reading performance
9	Nandyansah et al. (2020)	Picsar (Physics Augmented Reality) as a Learning Media to Practice Abstract Thinking Skills in Atomic Model
10	Yakubova et al. (2020)	Teaching students with ASD to solve fraction computations using a video modeling instructional package
11	Mena Araya (2020)	Critical.inking for Civic Life in Elementary Education: Combining Storytelling and Thinking Tools
12	Hwang et al. (2021)	Effects of a social regulation-based online learning framework on students’ learning achievements and behaviors in mathematics
13	Wang et al. (2022)	A Curation Activity-Based Self-Regulated Learning Promotion Approach as Scaffolding to Improving Learners’ Performance in STEM Courses
14	Kong and Liu (2023)	Supporting the Self-Regulated Learning of Primary School Students With a Performance-Based Assessment Platform for Programming Education
15	Ramdani et al. (2022)	Analysis of students’ self-regulated learning in terms of gender using blended learning-based laboratory inquiry teaching materials
16	Hidajat (2022)	The effects of E-learning based on a creatively cooperative method on student’s self-regulation ability in mathematics
17	Zhao et al. (2023)	Effects of a self-regulated-based gamified virtual reality system on students’ English learning performance and affection
18	Fitriyana et al. (2021)	The Influences of Hybrid Learning with Video Conference and “Chemondro-Game” on Students’ Self-Efficacy, Self-Regulated Learning, and Achievement toward Chemistry
19	Alserhan et al. (2023)	Personal Learning Environments: Modeling Students’ Self-Regulation Enhancement Through a Learning Management System Platform
20	Nacaroğlu and Bektaş (2023)	The effect of the flipped classroom model on gifted students’ self-regulation skills and academic achievement
21	Alhalafawy and Zaki (2022)	How Has Gamification Within Digital Platforms Affected Self-Regulated Learning Skills During the COVID-19 Pandemic? Mixed-Methods Research
22	Heaysman and Kramarski (2022)	Enhancing students’ metacognition, achievement and transfer between domains: Effects of the simulative “SRL-AIDE” parallel teacher–student program
23	Ikhsan et al. (2019)	The Effects of “Science-on-Web” Learning Media on Junior High School Students’ Learning Independency Levels and Learning Outcomes
24	Fößl et al. (2016)	A field study of a video supported seamless-learning-setting with elementary learners
25	T. L. Wong et al. (2018)	Promoting Self-Regulated Learning by Online Educational Resources
26	Huertas et al. (2015)	Effect of a computational scaffolding in the development of secondary students’ metacognitive skills
27	Mazzotti et al. (2013)	Effects of Multimedia Goal-Setting Instruction on Students’ Knowledge of the Self-Determined Learning Model of Instruction and Disruptive Behavior
28	Molenaar et al. (2012)	Dynamic scaffolding of socially regulated learning in a computer-based learning environment
29	Winters and Alexander (2011)	Peer collaboration: The relation of regulatory behaviors to learning with hypermedia
30	Meyer et al. (2010)	Improving literacy and metacognition with electronic portfolios: Teaching and learning with ePEARL

## Appendix B. Strategies the Interventions Aimed to Promote, by Type of Strategy

**Paper No.**	**Cognitive**	**Metacognitive**	**Motivational &** **Affective**	**Resource Management**
1	Problem-solving	Task selection, Self-assessment	Engagement	
2	Problem-solving	Task selection, Self-assessment	Engagement	
3	Reading comprehension	Goal setting, Learning monitoring, Self-assessment, Peer assessment	Self-efficacy, Intrinsic motivation	
4		Self-assessment		Learning pace management
5	Note-taking			
6	Note-taking	Planning, Monitoring, Self-assessment		Time management, Learning environment design, Help-seeking
7		Self-assessment, Strategy selection	Self-efficacy	
8	Note-taking			
9	Abstract scientific thinking			
10	Problem-solving, Mathematical thinking			
11	Problem-solving	Critical thinking		
12	Note-taking	Goal setting, Strategy selection	Self-confidence, Self-efficacy	Learning environment design
13	Information retrieval, Information organization			
14	Problem-solving			
15	Information organization, Information retrieval, Rehearsal	Planning, Goal setting	Self-confidence	Learning environment design, Help-seeking
16	Creative thinking, Mathematical thinking	Goal setting, Strategy selection, Self-assessment	Self-efficacy	Learning environment design, Help-seeking, Time management
17	Vocabulary acquisition	Goal setting, Monitoring, Strategy selection, Self-assessment	Engagement	Help-seeking, Time management
18	Elaboration	Reflection, Monitoring, Task evaluation	Self-efficacy	
19		Planning, Goal setting, Monitoring, Reflection, Evaluation	Self-efficacy, Effort	
20	Rehearsal, Elaboration, Information organization	Critical thinking, Self-assessment, Planning, Goal setting	Effort	Time management, Help-seeking
21	Rehearsal	Planning, Goal setting, Monitoring	Effort, Engagement	Help-seeking
22	Reading comprehension, Elaboration, Information organization	Monitoring, Strategy selection		
23			Intrinsic motivation, Self-confidence, Effort, Engagement	
24		Strategy selection, Planning, Goal setting	Engagement, Intrinsic motivation	Time management, Help-seeking
25		Planning, Goal setting, Monitoring, Task evaluation	Engagement, Intrinsic motivation	
26		Planning, Goal setting, Monitoring, Strategy selection, Self-assessment		
27		Planning, Goal setting, Monitoring, Self-assessment		
28	Writing, Elaboration	Planning, Goal setting, Monitoring		
29		Strategy selection, Monitoring, Engagement, Planning, Goal setting		
30		Planning, Goal setting, Self-assessment, Reflection, Strategy selection	Self-efficacy, Intrinsic motivation, Affect	
